# Risk of cancer and longest‐held occupations in Japanese workers: A multicenter hospital‐based case‐control study

**DOI:** 10.1002/cam4.2499

**Published:** 2019-08-12

**Authors:** Rena Kaneko, Masayoshi Zaitsu, Yuzuru Sato, Yasuki Kobayashi

**Affiliations:** ^1^ Department of Gastroenterology Kanto Rosai Hospital Kawasaki Japan; ^2^ Department of Public Health Graduate School of Medicine The University of Tokyo Tokyo Japan

**Keywords:** cancer, occupational activity, risk

## Abstract

**Objectives:**

Little is known about the risk of developing various cancers according to occupation and occupational physical activity.

**Methods:**

Using nationwide clinical inpatient data (1984‐2017) in Japan, we undertook a multicentered, matched case‐control study with regard to the risk of developing various cancers according to occupation and using patients admitted with fractures as controls. Using standardized national occupation and industrial classifications, we first identified the longest‐held job for each patient. Using sales workers as the reference group, odds ratios (ORs) and 95% confidence intervals (CIs) were estimated by conditional logistic regression, adjusted for age, admission period, and the admitting hospital, with smoking, alcohol consumption, and lifestyle diseases as covariates. The risk of high and low occupational physical activity was also estimated.

**Results:**

Across all occupations, a reduced risk for all common cancers among males was observed among those occupations associated with high physical activities, such as agriculture. People in these occupations tended to show a lower risk for most cancers, including, for example, prostate cancer (OR 0.58, 95% CI 0.45‐0.75) and lung cancer (OR 0.63, 95% CI 0.51‐0.76). For females, the breast cancer risk was low in women engaged in agriculture (OR 0.58, 95% CI 0.45‐0.75) and in those occupations with high levels of occupational physical activity (OR 0.58, 95% CI 0.52‐0.66).

**Conclusions:**

This study revealed differences in cancer risk among diverse occupations in Japan. Specifically, those occupations associated with high levels of physical activity may be associated with a decreased risk of cancer.

## Graphical abstract

The study revealed differences in cancer risk among diverse occupations in Japan. Specifically, those occupations associated with high levels of physical activity may be associated with a decreased risk of cancer.

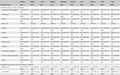

## 1 INTRODUCTION

Occupations, in particular, are a major social determinant of health.^1^ Working is generally recognized as having an effect on human health and well‐being; for example, specific occupations can cause harm in the form of malignant neoplasms.^2^, ^3^, ^4^

Globally, cancer is the second leading cause of death behind cardiovascular diseases, having caused over 8.7 million deaths in 2015.^5^ In Japan, cancer is the leading cause of death, with the total incidence of cancer in 2016 being estimated to be 867 408 (501 527 males and 365 881 females).^6^ In spite of this, it is thought that about half of all incidences of mortality from cancer in Japan are preventable.^7^

With regard to the role of occupation in the etiology of cancer, occupational exposure to carcinogens as well as occupation‐related lifestyle factors can add to the effective risk for cancer.^8^

Previous studies have investigated the association between occupation and cancer mortality.^9^, ^10^, ^11^ But, whether occupational physical activity is associated with a risk of cancer is unclear.^12^ In Japan, many working people spend a large proportion of their life in gainful employment. Unfortunately, stemming from a particular Japanese work culture, people's occupational activity may limit their ability to exercise. In several epidemiologic studies, an inverse association between recreational physical activity and cancer was established.^13^, ^14^, ^15^ In comparison, like “the physical activity paradox” for cardiovascular diseases, occupational and recreational physical activity has been associated with different effects on the risk of a particular cancer.^16^, ^17^

The aim of this study was to describe the cancer risk especially associated with occupation, paying attention to the level of occupational physical activity (OPA) in Japanese workers. Using nationwide, multicenter inpatient data, including individual‐level clinical data and occupational information, we examined the risk of cancer associated with each occupation.

### 1.1 Subjects

#### 1.1.1 Inpatient Clinico‐Occupational Database of Rosai Hospital Group

We used the Inpatient Clinico‐Occupational Database of Rosai Hospital Group (ICOD‐R), provided by the Japan Organization of Occupational Health and Safety (JOHAS), an independent administrative agency in Japan. Rosai Hospitals now comprise 33 hospitals, located from Hokkaido to Kyushu, in both rural and urban areas in Japan. The database contains medical chart information overseen by physicians, including a clinical history and diagnosis of current and past diseases, pathological information, treatments, and the outcome for every inpatient. The diagnoses are coded according to the International Statistical Classification of Diseases and Related Health Problems, 9th Revision (ICD‐9) or 10th Revision (ICD‐10). The ICOD‐R also includes an occupational history (current and past three jobs), information on lifestyle such as smoking and alcohol habits, and a history of lifestyle‐related diseases using interviews and questionnaires completed at the time of admission. Detailed occupational histories were coded using 3‐digit codes of the standardized national classification, the Japan Standard Occupational Classification and Japan Standard Industrial Classification, corresponding to the International Standard Industrial Classification and International Standard Occupational Classification. Written informed consent was obtained before patients completed questionnaires, and trained registrars were in charge of registering the data. Registrars are “health information managers”, qualified people in Japan. This is quite a unique feature of the database in Japan. The profiles of the inpatients are nationally representative because the Rosai Hospital Group has grown to cover all occupations since the establishment of the Rosai Hospital Group by the Ministry of Labour of Japan in 1949.

## 2 METHODS

The study was approved by the ethics committees of The University of Tokyo (No. 10891) and Kanto Rosai Hospital (No 2018‐11).

### 2.1 Case and control datasets

We obtained an anonymous dataset extracted from ICOD‐R with the permission of JOHAS, which included admissions to hospital between 1984 and 2017. We conducted a multicenter, hospital‐based matched case‐control study. With respect to selection cases and controls, we randomly sampled one control for each cancer case from cases of fractures of the arms and legs (ICD‐9, 810‐829 and ICD‐10, S40‐S99), matched for age, sex, period of admission date, and admitting hospital.

The cancer cases were difined by those patients with an inital diagnosis of cancer coded by ICD‐9 or ICD‐10. The cancer sites were selected according to national statistics in Japan,^6^, ^7^, ^18^ with the top most common cancer sites: prostate, breast, kidney, ureter, bladder, esophagus, stomach, liver, pancreas, colon, and lung. The prevalence of these cancers in our dataset was almost identical to that recorded by Japanese national statistics.^19^

### 2.2 Occupation categories and occupational physical activity

To evaluate the odds ratio of each occupation, we chose the longest‐held job of each patient from their occupational history.^11^, ^20^ Occupational categories were categorized into 12 categories according to the middle classification of the Japan Standard Occupational Classification of 2013. These categories were based on the International Standard Classification of Occupations. The twelve occupational categories were as follows: administrative and managerial; professional; clerical; sales; services; security; agriculture; manufacturing; transport; construction and mining; carrying, cleaning and packing; and others.

We also analyzed several additional categories. The occupational groups with high or low levels of OPA were defined based on information on their accelerometer‐derived occupational activities, modified by the National Health and Nutrition Examination Survey of 2003‐2004 (NHANES).^21^ High physical activity groups included agriculture, construction and mining, and carrying, cleaning and packing. The low activity groups included administrative and managerial, professional, and clerical.

### 2.3 Statistical analysis

Conditional regression analysis was used for the estimation of odd ratios (ORs), with 95% confidence intervals (95% CIs), for each occupational category in relation to the risk for each cancer. The sales workers group was selected as a reference category in accordance with a previous study.^22^ The risks for high and low OPA groups were estimated compared to the other remaining cases (eg, the low OPA group vs. a reference group of all cases except the low OPA group). We conducted separate analyses for males and females due to the etiology of cancer.^23^ Age was categorized every 5 years. The period of admission was categorized into four study periods (1984‐1990, 1991‐2000, 2001‐2010, 2011‐2017) and matched into pairs. Models were adjusted for age, sex, period of admission, and admission hospital. Smoking, consumption of alcohol, and lifestyle‐related diseases (hypertension, hyperlipidemia, hyperuricemia, diabetes mellitus, and obesity) were included as covariates. For smoking, we used the Brinkman Index: calculated as the number of cigarettes smoked per day multiplied by the number of years smoked. Alpha was set at 0.05, and all *P*‐values were two‐sided. All analyses were conducted using STATA/MP15.0 (Stata Corp LP).

## 3 RESULTS

The total number of inpatient cases registered in the ICOD‐R from 1984 to 2017 comprised 6 526 387. Of these, completed data were available for 6 309 852 cases, which included birthday, sex, ICD‐9, or ICD‐10 code, history of smoking and alcohol consumption. Of these cases, 4 186 750 were first‐time admissions of the initial admission cases, while occupational information was available for 1 843 672 cases. Cancers for the 57 913 cases included prostate, breast, kidney, ureter, bladder, esophagus, stomach, liver, pancreas, colon and lung. The demographics of each cancer are shown in Table 1.

**Table 1:** Sex and age distribution of all cancer cases and controls in each occupational category

Occupational category n=	ALL		Prostate		Breast		Kidney		Ureter		Bladder		Esophagus		Stomach		Liver		Pancreas		Colon		Lung	
	Case	Control	Case	Control	Case	Control	Case	Control	Case	Control	Case	Control	Case	Control	Case	Control	Case	Control	Case	Control	Case	Control	Case	Control
	57913	57913	3429	3429	5093	5093	1450	1450	1013	1013	5964	5964	2414	2414	11 839	11 839	2637	2637	3056	3056	12 470	12 470	8548	8548
ALL																								
n = (male:female)	41 957:15 956	41 955:15 956	3429:0	3428:0	75:5018	75:5018	1136:314	1136:314	828:185	828:185	5314:650	5314:650	2225:189	2225:189	9270:2569	9270:2569	2032:605	2032:605	2181:875	2181:875	8769:3701	8769:3701	6698:1850	6698:1850
Age(mean ± SD)	66.7 ± 11.5	66.8 ± 11.8	66.4 ± 8.8	66.4 ± 9.11	56.2 ± 12.8	56.4 ± 12.9	63.3 ± 11.9	63.4 ± 12.2	70.9 ± 9.6	71.0 ± 10.2	70.0 ± 10.7	70.1 ± 11.0	68.6 ± 9.33	68.6 ± 9.7	67.4 ± 11.3	67.4 ± 11.5	66.8 ± 10.8	66.8 ± 11.1	68.9 ± 10.4	68.9 ± 10.6	66.7 ± 11.5	66.7 ± 11.7	68.5 ± 10.3	68.5 ± 10.7
Administrative and managerial																								
n = (male:female)	2773:235	2587:248	225:0	226:0	3:48	7:61	84:4	49:6	63:4	62:5	376:19	342:14	155:2	158:3	570:42	595:35	130:12	117:13	145:11	139:16	591:63	524:65	398:30	368:30
Age(mean ± SD)	68.9 ± 10.0	71.1 ± 10.0	66.8 ± 8.3	68.7 ± 9.0	58.4 ± 12.7	64.7 ± 12.7	65.5 ± 11.1	68.3 ± 10.7	72.4 ± 10.2	73.7 ± 8.1	70.4 ± 10.3	73.3 ± 9.5	70.1 ± 7.8	71.0 ± 9.6	68.5 ± 10.0	70.9 ± 10.4	68.7 ± 10.2	69.9 ± 10.1	69.7 ± 9.9	72.5 ± 9.5	68.6 ± 10.2	70.6 ± 9.9	60.7 ± 9.3	72.3 ± 9.4
Professionals																								
n = (male:female)	4656:2263	4263:2507	501:0	336:0	19:910	12:994	167:42	133:52	93:16	83:24	621:92	567:78	201:7	232:27	998:306	948:372	214:66	204:86	248:103	226:94	1040:495	874:543	554:225	648:237
Age(mean ± SD)	64.5 ± 12.8	63.5 ± 13.1	64.5 ± 9.1	64.2 ± 9.5	53.3 ± 12.2	51.6 ± 12.3	59.8 ± 12.8	60.9 ± 13.6	70.4 ± 10.5	69.3 ± 11.9	69.4 ± 11.9	68.9 ± 12.0	68.4 ± 9.8	66.9 ± 10.1	65.8 ± 12.9	65.1 ± 12.7	67.2 ± 12.2	65.2 ± 11.2	68.2 ± 11.1	66.5 ± 11.4	64.9 ± 12.4	64.4 ± 12.4	67.1 ± 11.6	66.3 ± 11.5
Clerical																								
n = (male:female)	5788:3695	4549:3477	600:0	366:0	11:1466	5:1234	194:77	139:63	135:29	90:31	762:127	584:126	289:33	220:39	1272:513	998:563	269:115	210:111	274:170	230:168	1179:794	970:770	802:371	738:372
Age(mean ± SD)	64.3 ± 12.4	64.7 ± 12.6	65.3 ± 9.3	66.8 ± 9.2	53.9 ± 12.2	53.8 ± 11.3	61.7 ± 12.5	63.2 ± 11.9	69.5 ± 10.5	71.4 ± 10.9	69.2 ± 11.1	69.3 ± 11.7	67.4 ± 9.8	69.2 ± 10.9	65.9 ± 11.8	65.8 ± 12.3	65.7 ± 11.3	66.5 ± 11.8	68.2 ± 10.6	67.9 ± 10.9	65.2 ± 12.0	65.1 ± 12.3	66.6 ± 10.9	67.4 ± 11.2
Sales																								
n = (male:female)	4726:2127	4000:2042	367:0	343:0	5:683	10:619	139:38	125:40	90:34	85:23	626:95	494:92	254:23	212:26	1038:309	898:312	204:86	203:86	273:125	195:137	1070:505	824:493	660:222	611:221
Age(mean ± SD)	65.1 ± 11.7	65.8 ± 11.9	64.9 ± 8.5	65.5 ± 9.8	55.8 ± 13.2	57.0 ± 12.7	62.1 ± 11.8	62.4 ± 11.9	60.7 ± 10.7	70.5 ± 10.3	68.5 ± 11.2	69.2 ± 11.2	67.5 ± 10.0	67.3 ± 10.4	65.0 ± 11.5	66.3 ± 11.7	67.0 ± 10.2	66.9 ± 12.2	68.1 ± 10.1	67.5 ± 10.5	65.3 ± 11.6	66.0 ± 11.9	66.7 ± 10.3	67.4 ± 10.7
Services																								
n = (male:female)	2678:1476	1492:2663	100:0	121:0	5:750	1:812	37:55	43:59	40:35	23:31	156:111	201:105	115:50	87:22	301:423	332:432	81:136	69:105	78:135	73:136	318:625	324:620	239:358	218:347
Age(mean ± SD)	64.2 ± 11.1	65.2 ± 11.7	65.7 ± 8.0	64.9 ± 7.7	57.5 ± 11.8	58.5 ± 12.8	64.1 ± 10.8	63.4 ± 11.9	68.4 ± 9.2	68.9 ± 9.9	68.0:10.8	69.9 ± 10.7	65.2 ± 8.8	67.7 ± 10.2	65.5 ± 11.0	66.3 ± 11.1	65.7 ± 11.1	67.6 ± 9.9	65.5 ± 10.1	69.2 ± 10.0	65.1 ± 10.6	65.8 ± 11.3	66.0 ± 9.6	67.4 ± 10.1
Security																								
n = (male:female)	987:25	951:23	93:0	74:0	1:11	3:14	16:0	24:1	28:0	9:0	125:2	116:1	49:1	50:0	244:4	231:2	45:1	55:0	52:1	41:1	197:5	185:3	137:0	163:1
Age(mean ± SD)	67.8 ± 10.6	65.2 ± 11.6	64.9 ± 8.4	64.3 ± 9.1	55.8 ± 16.1	47.3 ± 13.9	66.0 ± 10.8	58.2 ± 13.9	69.8 ± 12.1	68.7 ± 9.9	69.3 ± 8.1	68.0 ± 11.7	68.5 ± 9.3	64.3 ± 9.2	67.6 ± 10.5	65.7 ± 11.4	68.8 ± 10.2	63.7 ± 10.2	68.6 ± 11.4	64.5 ± 11.6	68.3 ± 12.1	64.3 ± 12.6	68.6 ± 10.2	67.7 ± 10.1
Agriculture																								
n = (male:female)	2465:1270	3517:1375	183:0	274:0	3:153	3:228	55:22	81:22	46:22	83:21	299:69	458:67	128:20	185:19	614:308	792:268	114:57	167:67	147:100	200:115	464:346	682:366	412:173	592:202
Age(mean ± SD)	75.2 ± 9.9	74.2 ± 10.8	71.4 ± 9.3	71.9 ± 9.3	70.4 ± 13.4	68.9 ± 13.9	71.3 ± 10.6	69.1 ± 12.6	77.6 ± 8.3	76.9:9.4	77.7 ± 8.9	76.4 ± 9.4	73.8 ± 9.5	72.9 ± 9.6	75.8 ± 9.6	74.3 ± 10.8	72.1 ± 10.2	72.8 ± 10.6	76.1 ± 10.3	75.9 ± 10.2	75.7 ± 10.4	74.2 ± 11.1	75.9 ± 8.6	75.1 ± 10.1
Manufacturing																								
n = (male:female)	8824:2194	9197:2166	661:0	747:0	12:609	16:659	213:42	248:39	176:30	189:27	1160:72	1172:105	491:30	500:35	1941:401	1973:346	411:80	447:80	433:129	434:133	1747:513	1995:505	1579:283	1456:242
Age(mean ± SD)	67.7 ± 10.5	67.4 ± 10.7	67.0 ± 8.3	66.3 ± 8.7	60.0 ± 12.5	59,5 ± 12.5	64.7 ± 11.1	64.3 ± 11.9	71.1 ± 7.6	79.7 ± 10.0	70.7 ± 9.7	69.8 ± 10.6	68.8 ± 9.2	68.9 ± 8.6	67.6 ± 10.3	67.7 ± 10.6	67.5 ± 10.4	66.6 ± 10.4	68.9 ± 9.2	68.9 ± 10.1	66.9 ± 11.1	67.2 ± 10.7	68.6 ± 9.8	68.5 ± 9.9
Transport																								
n = (male:female)	4323:158	4422:150	297:0	374:0	4:62	8:52	65:2	120:3	60:1	89:1	526:4	547:5	209:1	229:1	990:24	979:18	249:2	228:3	240:10	230:10	907:37	898:32	756:15	720:25
Age(mean ± SD)	67.8 ± 9.9	66.5 ± 10.1	67.8 ± 7.3	65.0 ± 7.7	53.6 ± 13.5	58.3 ± 13.1	64.3 ± 10.4	61.8 ± 11.9	72.3 ± 7.7	68.9 ± 7.9	69.8 ± 10.1	68.6 ± 9.8	69.4 ± 8.3	66.9 ± 9.0	67.7 ± 9.7	66.7 ± 10.3	65.4 ± 10.6	63.8 ± 10.6	67.5 ± 9.7	67.2 ± 8.9	66.4 ± 10.1	65.9 ± 10.4	68.5 ± 9.4	67.7 ± 9.7
Construction and mining																								
n = (male:female)	4.515:121	5289:111	279:0	423:0	8:12	8:18	112:2	131:4	80:3	89:1	512:4	621:6	240:3	260:1	968:25	1169:25	248:7	241:7	221:12	307:6	936:31	1139:27	910:22	902:16
Age(mean ± SD)	68.1 ± 19.3	67.1 ± 10.5	67.6 ± 8.8	65.3 ± 8.7	62.6 ± 12.9	64.4 ± 7.8	63.4 ± 12.3	61.6 ± 10.4	70.4 ± 7.8	71.2 ± 9.3	69.4 ± 9.9	68.4 ± 10.8	68.6 ± 8.4	68.3 ± 9.1	67.7 ± 10.9	66.7 ± 10.5	64.7 ± 9.7	66.1 ± 10.8	69.2 ± 9.8	68.2 ± 10.4	68.0 ± 10.7	66.3 ± 11.2	69.1 ± 9.9	68.3 ± 10.2
Carrying, cleaning and packing																								
n = (male:female)	1329:1191	1608:1152	89:0	141:0	4:308	2:322	34:30	43:23	16:11	24:20	143:55	201:50	85:19	88:15	313:210	341:193	62:43	88:46	65:77	81:59	297:286	333:273	221:148	266:155
Age(mean ± SD)	66.1 ± 10.7	65.3 ± 10.9	68.6 ± 9.7	66.3 ± 7.9	60.1 ± 11.9	58.1 ± 12.8	63.2 ± 9.2	64.3 ± 11.9	68.2 ± 9.1	70.7 ± 10.5	68.4 ± 9.6	69.1 ± 10.7	67.8 ± 8.9	67.1 ± 7.9	66.3 ± 10.9	65.9 ± 10.4	64.7 ± 10.9	65.8 ± 10.4	68.5 ± 9.6	67.3 ± 9.2	66.6 ± 10.2	64.4 ± 10.5	67.8 ± 9.7	66.9 ± 9.9
Other																								
n = (male:female)	103:16	80:19	1:0	4:0	0:6	0:5	0:0	0:1	1:0	3:0	8:0	11:1	9:0	4:1	21:4	14:3	5:0	3:1	5:2	5:0	23:1	20:5	30:3	16:2
Age(mean ± SD)	77.5 ± 9.3	79.4 ± 9.9	—	78.5 ± 2.5	63.3 ± 13.8	64.4 ± 17.7	—	—	—	69.7 ± 10.2	78.1 ± 7.6	85.1 ± 4.1	74.0 ± 12.8	85.2 ± 2.9	78.9 ± 9.0	78.8 ± 9.1	70.0 ± 8.1	82.8 ± 3.8	81.6 ± 8.2	81.2 ± 7.0	77.3 ± 8.9	76.6 ± 9.8	78.6 ± 6.7	82.6 ± 10.6
*Occupational physical activity group*																								
High activity†																								
n = (male:female)	8309:2582	10 414:2638	551:0	838:0	15:473	13:568	201:54	255:49	142:36	196:42	954:128	1280:123	453:42	533:35	1895:543	2302:486	424:107	496:120	433:180	588:180	1697:663	2154:666	1543:343	1760:373
Age(mean ± SD)	60.1 ± 10.9	69.4 ± 11.4	69.1 ± 9.3	67.6 ± 9.4	63.5 ± 13.3	62.7 ± 14.1	65.8 ± 11.6	64.7 ± 11.9	72.9 ± 9.0	73.6 ± 9.9	72.1 ± 10.3	71.5 ± 10.9	69.9 ± 9.1	69.7 ± 9.4	60.4 ± 11.3	69.4 ± 11.3	67.1 ± 10.7	68.6 ± 11.1	71.8 ± 10.6	71.2 ± 10.8	70.3 ± 11.2	68.9 ± 11.8	70.9 ± 10.1	70.6 ± 10.7
Low activity ‡																								
n = (male:female)	13 217:6193	11 399:6232	1359:0	928:0	33:2424	24:2289	445:123	321:122	291:49	235:60	1759:238	1493:218	645:42	610:69	2840:861	2541:970	613:193	531:210	667:284	595:278	2810:1352	2368:1378	1754:626	1754:639
Age(mean ± SD)	65.1 ± 12.3	65.3 ± 12.7	65.3 ± 9.1	66.4 ± 9.5	53.8 ± 12.2	53.2 ± 12.0	61.5 ± 12.6	64.9 ± 12.7	70.4 ± 10.5	71.2 ± 10.8	69.4 ± 11.3	69.9 ± 11.6	68.3 ± 9.4	68.7 ± 10.4	66.3 ± 11.8	66.5 ± 12.3	66.8 ± 11.5	66.6 ± 11.4	68.5 ± 10.7	68.2 ± 11.0	65.6 ± 11.9	65.7 ± 12.2	67.5 ± 10.9	67.8 ± 11.2

Note

When only one case was included in a column, age was not indicated to avoid any chance of patient identification.

SD, standard deviation

^†^ High activity group included agriculture, construction and mining, and carrying, cleaning and packing.

^‡^ Low activity group included administrative and managerial; professional and clerical support.

The average age of breast cancer patients was the lowest of all cancer sites (56.2 ± 12.8 years; Table 1). The mean age of ureter and bladder cancer patients was over 70 years (70.9 ± 9.6, 70.0 ± 10.7 years, respectively). With regard to occupational sites, the mean age of cancer patients working in agriculture was from 69.1 ± 12.6‐77.7 ± 8.9 years depending on cancer sites, which was the highest for all the occupational categories. The mean age of patients in the high OPA group (63.5 ± 13.3‐72.9 ± 9.0 years) was greater than that of patients in the low OPA group (53.8 ± 12.2‐70.4 ± 10.5 years), except for liver cancer.

For males, the sample size for the regression analysis was large enough to handle using nine variables. For females, the number of cases about OPA category was large enough, though each occupational category with several cancer cases was under 90 cases (number of variables times 10).

Table 2 shows the Brinkman Index, amount of alcohol consumed and number of patients with a history of lifestyle‐related diseases for each industrial category. The number of patients who regularly smoked or took alcohol tended to be fewer in agriculture.

**Table 2:** Distribution of life‐related diseases for each occupational category

Occupational category	Brinkman Index‡		Alcohol (g/day)		Hypertension		Hyperlypidemia		Hyperuricemia		Diabetes		Obesity	
	Median (IQR†25%:75%)		Median (IQR†25%:75%)		n = (%)		n = (%)		n = (%)		n = (%)		n = (%)	
	Case	Control	Case	Control	Case	Control	Case	Control	Case	Control	Case	Control	Case	Control
All	410 (0:840)	250 (0:740)	23.5 (0:75.0)	0 (0:600)	20 099 (34.7)	19 461 (33.6)	6726 (11.6)	6866 (11.8)	1769 (3.1)	1778 (3.1)	8683 (15.0)	9206 (15.9)	7451 (12.9)	6912 (11.9)
Administrative and managerial	640 (200:1020)	460 (0:920)	48.0 (0:90.0)	32.4 (0:82.0)	1224 (40.7)	1080 (38.1)	433 (14.4)	470 (16.6)	173 (5.7)	121 (4.3)	602 (20.0)	645 (22.8)	534 (17.7)	495 (17.5)
Professionals	240 (0:700)	65 (0:540)	80.0 (0:60.0)	0 (0:40.0)	2292 (33.1)	2075 (30.6)	1134 (16.4)	1162 (17.2)	283 (4.1)	286 (4.2)	990 (14.3)	869 (12.8)	1105 (15.9)	946 (13.9)
Clerical	225 (0:740)	20 (0:540)	55.0 (0:62.5)	0 (0:40.0)	3059 (32.3)	2591 (32.3)	1422 (15.0)	1325 (16.5)	308 (3.3)	231 (2.9)	1308 (13.8)	1038 (12.9)	1404 (14.8)	1150 (14.3)
Sales	400 (0:840)	220 (0:720)	23.0 (0:74.0)	30.0 (0:60.0)	2349 (34.3)	2048 (33.9)	821 (11.9)	703 (11.6)	247 (3.6)	250 (4.1)	1104 (16.1)	1059 (17.5)	971 (14.2)	773 (12.8)
Services	100 (0:620)	0 (0:450)	0 (0:47.0)	0 (0:32.0)	1323 (31.9)	1372 (33.0)	422 (10.2)	461 (11.1)	67 (1.6)	77 (1.9)	498 (12.0)	670 (16.1)	509 (12.3)	504 (12.1)
Security	600 (200:900)	480 (3:900)	50.5 (0:86.0)	28.0 (0:760)	408 (40.3)	332 (34.1)	154 (15.2)	169 (17.4)	43 (4.3)	16 (1.6)	166 (16.4)	184 (18.9)	176 (17.4)	187 (19.2)
Agriculture	198 (0:840)	25 (0:775)	0 (0:70.0)	0 (0:58.0)	1455 (38.9)	1730 (35.4)	282 (7.6)	298 (6.1)	69 (1.9)	51 (1.0)	527 (14.1)	770 (15.7)	246 (6.6)	258 (5.3)
Manufacturing	480 (0:855)	330 (0:760)	30.0 (0:76.0)	90.0 (0:64.0)	3751 (34.1)	3986 (35.1)	1027 (9.3)	1195 (10.5)	283 (2.6	330 (2.9)	1577 (14.3	1710 (15.1)	1273 (11.6)	1142 (10.1)
Transport	705 (300:1000)	520 (70:900)	54.0 (0:90.0)	40.0 (0:80.0)	1696 (37.9)	1604 (35.1)	423 (9.4)	471 (10.3)	127 (2.8)	197 (4.3)	802 (17.9)	1029 (22.5)	539 (12.0)	519 (12.9)
Construction and mining	675 (300:900)	560 (85:880)	54.0 (0:90.0)	36.0 (0:80.0)	1673 (36.1)	1783 (33.0)	339 (7.3)	362 (6.7)	115 (2.5)	147 (2.7)	756 (16.3)	684 (16.0)	404 (8.7)	515 (9.5)
Carrying, cleaning and packing	225 (0:740)	120 (0:600)	0 (0:60.0)	0 (0:46.0)	822 (32.7)	818 (29.6)	260 (10.3)	247 (8.9)	50 (2.0)	69 (2.5)	338 (13.4)	348 (12.6)	286 (11.4)	346 (12.5)
Other	580 (154:940)	560 (0:1140)	44.0 (0:85.5)	60.0 (0:104.0)	47 (39.5)	42 (42.4)	9 (7.6)	3 (3.0)	4 (3.4)	3 (3.0)	15 (12.6)	20 (20.2)	6 (5.0)	5 (5.1)
Occupational physical activity group														
High activity group§	490 (0:900)	350 (0:800)	28.0 (0:80.0)	0 (0:68.0)	3950 (36.3)	4331 (33.2)	881 (8.1)	907 (6.9)	234 (2.2)	267 (2.1)	1621 (14.9)	1982 (15.2)	936 (8.6)	1119 (8.6)
Low activity group ¶	370 (0:800)	110 (0:600)	20.0 (0:70.0)	0 (0:50.0)	3614 (34.6)	5746 (32.6)	1537 (14.7)	2957 (16.8)	391 (3.8)	638 (3.6)	1673 (16.0)	2552 (14.5)	1629 (15.6)	2591 (14.7)

^†^ IQR:Interquartile range.

^‡^ Brinkman Index: the number of cigarettes smoked per day multiplied by the number of years smoked.

^§^ High activity group included agriculture; construction and mining, and carrying, cleaning and packing.

^¶^ Low activity group included administrative and managerial; professional and clerical support.

Agriculture was significantly associated with reduced risks for most cancers in males (Table 3). In this category, the adjusted ORs were low, with a significant difference observed for all cancer sites. They ranged from 0.46 (95% CI 0.27‐0.78) for ureter carcinoma to 0.63 (95% CI 0.55‐0.74) for carcinoma in the stomach (0.63 95% CI 0.46‐0.88), liver (0.63 95% CI 0.46‐0.88), and lung (0.63 95% CI 0.51‐0.76). The high OPA group also tended to be associated with a lower risk for all cancers, ranging from 0.58 (95% CI 0.52‐0.66) for prostate cancer to 0.79 (95% CI 0.72‐0.86) for lung cancer. However, no obvious effect except for breast cancer was detected in female cases. The odds of breast cancer for those in agriculture (0.58 95% CI 0.45‐0.75) and the high OPA group (0.58 95% CI 0.52‐0.66) were significantly lower (Table 4).

**Table 3:** Odds ratio of each occupational category to cancer in males

Numbers of cases	Prostate	Kidney	Ureter	Bladder	Esophagus	Stomach	Liver	Pancreas	Colon	Lung
	3429	1136	828	5314	2225	9270	2032	2181	8769	6698
Occupational category (sales as a reference)										
Administrative and managerial	1.02 (0.81‐1.92)	1.45 (0.93‐2.24)	0.99 (0.61‐1.61)	0.84 (0.69‐1.02)	0.81 (0.59‐1.09)	0.81 (0.69‐0.93)	1.07 (0.77‐1.49)	0.69 (0.50‐0.94)	0.87 (0.75‐1.02)	0.96 (0.79‐1.19)
	.862	.099	.975	.085	.172	.005**	.686	.017*	.082	.751
Professionals	1.32 (1.07‐1.63)	1.12 (0.78‐1.59)	1.09 (0.70‐1.69)	0.90 (0.76‐1.07)	0.68 (0.52‐0.91)	0.96 (0.84‐1.09)	1.10 (0.83‐1.46)	0.81 (0.62‐1.05)	0.94 (0.82‐1.070	0.90 (0.76‐1.07)
	.008**	.545	.693	.247	.008**	.501	.500	.120	.344	.261
Clerical	1.45 (1.19‐1.78)	1.15 (0.81‐1.63)	1.46 (0.96‐2.22)	1.06 (0.90‐1.26)	1.00 (0.76‐1.32)	1.12 (0.98‐1.26)	1.29 (0.98‐1.69)	0.85 (0.65‐1.09)	0.95 (0.84‐1.08)	1.11 (0.94‐1.32)
	.000**	.425	.075	.458	.972	.086	.069	.209	.434	.206
Sales (ref)	1.00 (ref)	1.00 (ref)	1.00 (ref)	1.00 (ref)	1.00 (ref)	1.00 (ref)	1.00 (ref)	1.00 (ref)	1.00 (ref)	1.00 (ref)
	—	—	—	—	—	—	—	—	—	—
Services	0.73 (0.54‐1.00)	0.74 (0.44‐1.24)	1.59 (0.85‐2.96)	0.65 (0.51‐0.83)	0.99 (0.69‐1.42)	0.80 (0.67‐0.96)	1.14 (0.78‐1.69)	0.78 (0.53‐1.14)	0.77 (0.64‐0.93)	1.14 (0.89‐1.45)
	.051	.255	.145	.001**	.983	.019*	.496	.194	.006**	.289
Security	1.10 (0.78‐1.55)	0.55 (0.27‐1.12)	2.91 (1.17‐7.26)	0.81 (0.61‐1.08)	0.79 (0.50‐1.26)	0.89 (0.72‐1.09)	0.81 (0.52‐1.29)	0.85 (0.54‐1.35)	0.83 (0.67‐1.03)	0.80 (0.61‐1.07)
	.578	.101	.022*	.158	.331	.282	.397	.499	.103	.131
Agriculture	0.58 (0.45‐0.75)	0.58 (0.37‐0.93)	0.46 (0.27‐0.78)	0.49 (0.40‐0.61)	0.51 (0.37‐0.70)	0.63 (0.55‐0.74)	0.63 (0.46‐0.88)	0.49 (0.36‐0.66)	0.49 (0.42‐0.58)	0.63 (0.51‐0.76)
	.000**	.022*	.004**	.000**	.000**	.000**	.006**	.000**	.000**	.000**
Manufacturing	0.77 (0.64‐0.93)	0.77 (0.55‐1.06)	0.83 (0.56‐1.23)	0.79 (0.69‐0.92)	0.76 (0.61‐0.97)	0.85 (0.76‐0.96)	0.87 (0.69‐1.12)	0.67 (0.53‐0.85)	0.67 (0.59‐0.75)	1.05 (0.91‐1.22)
	.009**	.106	.362	.002**	.029	.006**	.281	.001**	.000**	.478
Transport	0.73 (0.58‐0.91)	0.57 (0.38‐0.85)	0.58 (0.37‐0.94)	0.74 (0.62‐0.88)	0.69 (0.53‐0.93)	0.85 (0.75‐0.78)	0.98 (0.74‐1.29)	0.72 (0.55‐0.94)	0.76 (0.67‐0.87)	0.93 (0.78‐1.10)
	.005**	.006**	.026**	.001**	.012*	.000**	.879	.014**	.000**	.403
Construction and mining	0.58 (0.47‐0.73)	0.74 (0.52‐1.07)	0.81 (0.52‐1.26)	0.63 (0.53‐0.75)	0.66 (0.50‐0.87)	0.68 (0.61‐0.78)	0.98 (0.75‐1.29)	0.49 (0.38‐0.64)	0.62 (0.54‐0.70)	0.92 (0.78‐1.08)
	.000**	.114	.343	.000**	.003**	.000**	.891	.000**	.000**	.328
Carrying, cleaning and packing	0.58 (0.43‐0.80)	0.71 (0.42‐1.20)	0.58 (0.28‐1.21)	0.57 (0.44‐0.73)	0.81 (0.55‐1.19)	0.77 (0.64‐0.92)	0.74 (0.50‐1.08)	0.52 (0.35‐0.77)	0.69 (0.57‐0.82)	0.71 (0.56‐0.89)
	.001**	.198	.149	.000**	.287	.005**	.126	.000**	.000**	.003**
Other	0.20 (0.02‐1.85)	N/A	0.11 (0.01‐1.19)	0.51 (0.19‐1.29)	1.57 (0.39‐6.37)	1.20 (0.60‐2.42)	1.45 (0.33‐6.40)	0.51 (0.12‐2.11)	0.80 (0.43‐1.48)	2.28 (1.13‐4.61)
	.156	N/A	.070	.157	.527	.600	.619	.351	.481	.021*
Occupational activity group (the other workers as a reference)										
High activity†	0.58 (0.52‐0.66)	0.74 (0.60‐0.93)	0.65 (0.50‐0.83)	0.67 (0.61‐0.73)	0.77 (0.65‐0.90)	0.75 (0.69‐0.80)	0.80 (0.69‐0.94)	0.65 (0.56‐0.75)	0.72 (0.67‐0.77)	0.79 (0.72‐0.86)
	.000**	.009**	.001**	.000**	.001**	.000**	.000**	.000**	.000**	.000**
Low activity‡	1.74 (0.57‐1.93)	1.60 (1.33‐1.93)	1.45 (1.16‐1.79)	1.29 (1.18‐1.41)	1.09 (0.94‐1.26)	1.21 (1.13‐1.29)	1.28 (1.11‐1.48)	1.19 (1.04‐1.37)	1.03 (1.22‐1.39)	1.08 (0.99‐1.17)
	.000**	.000**	.001**	.000**	.215	.000**	.001**	.013*	.000**	.104

Note

Odds ratios were estimated by conditional logistic regression matched for age, sex, admission period, and admitting hospital.

The upper row shows odds ratios (95% confidence interval) against sales workers as a reference (ref).

The lower row shows *P*‐values of <.01** or <.05* were considered to be statistically significant

NA: Data was not available for a number of cases, making this category too small.

^†^ High activity group included agriculture, construction and mining, and carrying, cleaning and packing

^‡^ Low activity group included administrative and managerial, and professional and clerical support.

**Table 4:** Odds ratio of each occupational category to cancer in females

Numbers of cases	Breast	Kidney	Ureter	Bladder	Esophagus	Stomach	Liver	Pancreas	Colon	Lung
	5018	314	185	650	189	2569	605	875	3701	1850
Occupational category (sales as a reference)										
Administrative and managerial	0.68 (0.17‐2.70)	0.68 (0.17‐2.74)	0.52 (0.12‐2.25)	1.32 (0.59‐2.97)	5.68 (0.62‐52.11)	1.13 (0.69‐1.83)	0.77 (0.31‐1.94)	0.78 (0.34‐1.78)	0.95 (0.66‐1.37)	0.97 (0.55‐1.71)
	.862	.592	.382	.498	.124	.629	.578	.550	.798	.911
Professionals	1.32 (1.07‐1.63)	0.89 (0.47‐1.68)	0.34 (0.13‐0.86)	1.15 (0.75‐1.77)	0.21 (0.05‐0.80)	0.84 (0.67‐1.05)	0.83 (0.53‐0.1.29)	1.29 (0.89‐1.89)	0.89 (0.75‐1.06)	0.97 (0.74‐1.27)
	.008**	.716	.024*	.532	.023*	.123	.413	.186	.183	.836
Clerical	1.45 (1.19‐1.78)	1.43 (0.78‐2.63)	0.49 (0.22‐1.10)	1.03 (0.69‐1.55)	1.23 (0.55‐2.73)	0.91 (0.74‐1.11)	1.01 (0.67‐1.54)	1.17 (0.84‐1.65)	1.00 (0.86‐1.18)	1.09 (0.86‐1.39)
	.000**	.252	.084	.871	.610	.345	.946	.356	.958	.484
Sales (ref)	1.00 (ref)	1.00 (ref)	1.00 (ref)	1.00 (ref)	1.00 (ref)	1.00 (ref)	1.00 (ref)	1.00 (ref)	1.00 (ref)	1.00 (ref)
	—	—	—	—	—	—	—	—	—	—
Services	0.73 (0.54‐1.00)	1.01 (0.55‐1.87)	0.69 (0.33‐1.49)	0.93 (0.62‐1.39)	3.04 (1.21‐7.65)	0.97 (0.69‐1.19)	1.19 (0.79‐1.81)	1.09 (0.77‐1.56)	0.97 (0.82‐1.15)	0.92 (0.72‐1.18)
	.051	.972	.350	.728	.018*	.791	.409	.612	.738	.527
Security	1.10 (0.78‐1.55)	N/A	N/A	2.59 (0.23‐29.71)	N/A	2.41 (0.43‐13.41)	N/A	0.99 (0.06‐16.37)	1.59 (0.38‐6.73)	N/A
	.578	N/A	N/A	.443	N/A	.315	N/A	.997	.528	N/A
Agriculture	0.58 (0.45‐0.75)	0.96 (0.43‐2.19)	0.61 (0.25‐1.52)	1.01 (0.60‐1.68)	2.18 (0.77‐6.21)	1.26 (0.98‐1.62)	0.83 (0.51‐1.35)	0.94 (0.62‐1.43)	0.93 (0.75‐1.15)	0.91 (0.67‐1.24)
	.000**	.935	.290	.981	.143	.062	.446	.788	.500	.559
Manufacturing	0.77 (0.64‐0.93)	1.02 (0.53‐1.97)	0.78 (0.35‐1.77)	0.70 (0.45‐1.08)	1.18 (0.51‐2.73)	1.21 (0.98‐1.51)	1.00 (0.64‐1.58)	1.11 (0.77‐1.59)	0.99 (0.83‐1.19)	1.21 (0.93‐1.58)
	.009**	.943	.555	.107	.700	.077	.995	.576	.921	.156
Transport	0.73 (0.58‐0.91)	0.68 (0.10‐4.57)	0.99 (0.04‐23.94)	0.61 (0.15‐2.53)	33.9 (0.77‐1487.95)	1.26 (0.66‐2.41)	0.63 (0.09‐4.23)	1.09 (0.43‐2.77)	1.15 (0.69‐1.88)	0.48 (0.24‐0.97)
	.005**	.693	.997	.501	.068	.477	.637	.857	.590	.041*
Construction and mining	0.58 (0.47‐0.73)	0.43 (0.06‐2.72)	2.73 (0.24‐31.20)	0.73 (0.19‐2.73)	21.06 (0.78‐569.29)	1.06 (0.58‐1.91)	0.72 (0.24‐2.16)	2.15 (0.77‐5.98)	1.11 (0.69‐1.88)	1.21 (0.61‐2.42)
	.000**	.368	.418	.639	.07	.853	.555	.144	.590	.577
Carrying, cleaning and packing	0.58 (0.43‐0.80)	1.34 (0.63‐2.86)	0.31 (0.12‐0.78)	1.10 (0.67‐1.80)	1.78 (0.66‐4.82)	1.09 (0.85‐1.42)	0.93 (0.54‐1.60)	1.42 (0.92‐2.21)	1.11 (0.64‐1.91)	0.95 (0.69‐1.29)
	.001**	.448	.014*	.703	.257	.469	.785	.116	.901	.741
Other	0.20 (0.02‐1.85)	N/A	N/A	N/A	N/A	1.46 (0.31‐6.81)	N/A	N/A	0.20 (0.02‐1.75)	2.07 (0.28‐15.17)
	.156	N/A	N/A	N/A	N/A	.629	N/A	N/A	.147	.472
Occupational activity group (the other workers as a reference)										
High activity†	0.58 (0.52‐0.66)	1.08 (0.67‐1.73)	0.77 (0.46‐1.29)	1.11 (0.82‐1.49)	1.56 (0.87‐2.79)	1.18 (1.02‐1.37)	0.85 (0.63‐1.17)	1.06 (0.82‐1.38)	1.00 (0.88‐1.14)	0.92 (0.78‐1.11)
	.000**	.742	.328	.510	.132	.026*	.321	.634	.964	.419
Low activitys‡	1.74 (0.57‐1.93)	1.09 (0.75‐1.57)	0.65 (0.39‐1.09)	1.20 (0.93‐1.55)	0.55 (0.34‐0.92)	0.82 (0.72‐0.92)	0.91 (0.70‐1.17)	1.08 (0.88‐1.33)	0.97 (0.88‐1.07)	1.05 (0.91‐1.22)
	.000**	.651	.102	.151	.022*	.001**	.448	.454	.531	.493

Note

Odds ratios were estimated by conditional logistic regression matched for age, sex, admission period, and admitting hospital.

The upper row shows odds ratios (95% confidence interval) against sales workers as a reference (ref).

The lower row shows a *P*‐value of <.01** or <.05* were considered to be statistically significant

The upper row shows odds ratios (95% confidence interval) against sales workers as a reference (ref).

^†^ High activity group included agriculture, construction and mining, and carrying, cleaning and packing.

^‡^ Low activity group included administrative and managerial, professional and clerical support.

As a whole in males, an overall reduced risk for all cancers was associated with those occupations related to high OPA. In females, a reduced risk for breast cancer showed a similar tendency: an association with occupations characterized by high OPA.

## 4 DISCUSSION

### 4.1 Change of occupational risk in a historical transition

In general, workers are engaged in their jobs for about 40 hours per week, which means that they spend one‐fourth of their time in job‐related activities. Moreover, many Japanese companies have conventionally adopted the lifetime employment system: a promise by a company to an employee that they will have a job for their whole working life, as is customary in Japanese society. Therefore, most workers’ lifetime physical activity in Japan can be defined by an OPA.

In Japan, one of the main changes observed in the employment sector in recent years has been the decline in the proportion of the population working in agriculture. In comparison, the proportion of workers engaged in the tertiary sector, including sales and services, is increasing rapidly. As a result, physical activity derived from an occupation has tended to diminish. Such major changes have been observed from the late twentieth century onwards, and have coincided with the growth of the welfare state and the increasing urbanization of the population.

Another important change has been the increase in female labor in the workplace. Women have been released from a life of domestic servitude in the home and have become increasingly engaged in the workforce.

Such factors outlined above may have strongly influenced the occupational factors associated with national cancer incidence and mortality in Japan.

### 4.2 Agriculture

Previous studies indicated that agricultural workers represent a unique population, possibly due to differences in lifestyle or their exposure to risky environmental hazards. Such workers deal with many potential hazards that include pesticides, chemical and biological agents, and the operation of heavy equipment. However, a lower prevalence of smoking plus high occupational activity has been reported^24^, ^25^ for this sector and this may have influenced the low mortality and morbidity rates from cancers observed among farmers.^25^

In a previous large Canadian cohort study linked with cancer registry records, hazard ratios of the agriculture sector for lung, colon, bladder, kidney and liver cancers were found to be significantly lower than for other occupational workers.^25^ The risk reduction observed for kidney, bladder and colon cancers may be because of the working conditions of agricultural workers that involve high physical activity, a recognized modifiable risk factor for such cancers.^26^ An analysis from a Spanish population‐based case‐control study revealed no significant association between male farmers and pancreatic cancer.^27^ In comparison, for prostate cancer, established risk factors were age, ethnicity, and a positive family history of prostate cancer when comparing farmers to non‐farmers.^28^

Farmers tend to not have an occupational retirement age since they are limited by physical strength and health, even though the normal time for retirement in Japan is between 60 and 65 years. In our study, although farmers were somewhat older than those employed in other occupations, the risk was significantly lower for all sites of cancer than that of other occupations. This difference explains how a risk‐reductive process may exist in the agricultural sector.

In Japan in the middle of the twentieth century, about 18 million people were employed in agriculture, then one of the largest occupational groups. Since the economic significance of the agricultural sector has declined in parallel with an increase in the service sector, the proportion of people employed in this sector decreased to 6.3 million by the end of the last century.^29^ Thus, the particular reasons for cancer risk reductions in agriculture must be understood against a background of a decline and graying of the farming population.

### 4.3 High occupational physical activity and a paradox

In this study, we found significant associations between high levels of OPA and the risk of common cancers in males. This suggests that OPA may have some impact on the risk of developing cancer.

The present study is in agreement with previous studies reporting an association between high physical activity (including both occupational and leisure time) and cancer occurrence.^12^, ^13^, ^14^ Although the mechanism is as yet undefined, both hormonal and nonhormonal causal relationships between physical activity and cancer are suspected.^26^ An association between physical activity and hyperinsulinemia, inflammation, and immune disorders are potential nonhormonal etiologies of cancer.^30^, ^31^ In comparison, in an example of a hormonal factor involved in the development of cancer, it was shown that physical activity helped reduce levels of cancer‐relevant biomarkers such as estradiol by preventing any above‐normal weight gain.^32^, ^33^ Since, cancers are known to be obesity‐related, it is unclear whether physical activity or obesity is the key to carcinogenesis.^26^

It has also been reported that only recreational physical but not occupational activity diminished the cancer risk.^16^ The so‐called “physical activity health paradox” may be due to the difference in characteristics of recreational and occupational physical activities.^34^ This phenomenon has been mainly discussed in relation to cardiovascular disease,^17^, ^35^ but with regard to lung cancer, the same tendency has been reported.^16^

In this study, the physical activity health paradox was not observed since the high OPA group showed low rates of obesity and a low risk of cancer. One may speculate that the effect of a low percentage of obesity due to high OPA may cause a reduction in risk rather than being an unhealthy result due to a specific OPA. The interwoven complexity of physical activity, obesity, and cellular pathways in cancer is yet to be disentangled. However, it is plausible that sedentary behavior may contribute to carcinogenesis.

### 4.4 Strengths and limitations

As far as we are aware, we are the first to investigate the association between occupations, especially OPA, and the risk of developing common cancers (not mortality) in Japan. This study is also one of the largest studies on the risk of developing cancer reported in the country. The particular strengths of this study include accurate diagnoses, which were directly extracted from medical charts in contrast to the less accurate diagnoses from claims data as done in other studies.^36^ The exposure to a specific OPA was estimated, with quantification based on the amount of physical activity, measured with an accelerometer categorized more specifically by NHANES.^21^

However, despite this, several limitations may still exist. First, the content of ICOD‐R may have been flawed. Other factors relevant to the study, such as the presence of pathogenic organisms (ie, *Helicobacter pylori* in stomach cancer, hepatitis virus in liver cancer) or socioeconomic status （ie, amount of income, educational attainment） could not be evaluated due to the limitations of the data. In addition, our data were not designed to detect occupational exposure to carcinogens or the high risks associated with specific occupational situations.^37^ But, with regard to several established risk factors, low physical activity still remained a risk for several cancers.^12^, ^14^, ^38^ Although our findings do not elucidate a specific relationship between OPA and cancer, the associations identified in this study may be implied.

Because ICOD‐R is not a relevant population‐based database, the hospital‐based case‐controls we used may have had a selection bias. In addition, one‐third of the missing information within an occupation may amplify any selection bias even though all available factors were included as covariates in statistics. This problem arose because the return of occupational data from patients was not enforced because of concerns about the protection of patients’ privacy. This has the effect of making any selection bias stronger and may have affected results that were insignificant for each occupational risk in females. An information bias existed in terms of misclassifications in occupational categories because data recall was from disease onset. Confirmation of this can be found in a previous study since occupational profiles in this database are nationally representative.^19^ These are issues that need to be resolved in future in order to increase the accuracy of the dataset.

Second, a screening bias existed within the results. Though, medical checkup systems are widespread and covered on a national basis by medical insurance in Japan, disparities still exist in terms of opportunities for undergoing a medical examination among occupations and residential areas. Agriculture workers tend to work in self‐owned businesses and live in rural areas, so that the chance of diagnosing carcinoma is likely lower than for other occupations. We could not adjust such an inequality in the chance to undergo screening even though adjustments for areas were undertaken.

Finally, evaluating occupational risk using the longest‐held job may have led to a biased influence. The identified occupations used in this study were those in which individuals were mainly engaged in throughout their lifetime. On this point, this is a more accurate assessment of occupational risk than choosing the occupation of the patient at the time of death.^11^, ^20^ However, this may not always be the most relevant for deducing cancer risk. Considering the incubation time from exposure to an OPA adds to the risk of carcinogenicity, in any future studies using whole occupations over a lifetime and the time lag to developing cancer must be estimated.

More detailed studies in future will evaluate the occupational aspects of cancer causal relationships in an increasingly statistical manner.

## 5 CONCLUSION

We have documented occupational inequalities in the risk of developing various cancers in Japanese workers. High levels of occupational physical activity are associated with a decreased risk of various cancers in men and decreased breast cancer in women. Further research on occupational physical activity and cancer risk in another large population may lead to an improvement in the health of the general population.

## CONFLICTS OF INTEREST

None.

## AUTHOR CONTRIBUTIONS

Rena Kaneko: Funding acquisition, conceptualization, resources, formal analysis, writing—original draft, review and editing. Masayoshi Zaitsu: Review and editing. Yuzuru Sato: Funding acquisition, review and editing. Yasuki Kobayashi: Funding acquisition, supervision, review and editing.

## INFORMED CONSENT

This study involved a retrospective analysis of data that had already been obtained through a national survey. As we did not use any personally identifiable information based on existing regulations in Japan, personal informed consent was not required.

## Data Availability

The data that support the findings of this study are available on request from the corresponding author. The data are not publicly available due to privacy or ethical restrictions.
